# Sewage sludge application enhances soil properties and rice growth in a salt-affected mudflat soil

**DOI:** 10.1038/s41598-020-80358-2

**Published:** 2021-01-14

**Authors:** Yuhua Shan, Min Lv, Wengang Zuo, Zehui Tang, Cheng Ding, Zhixuan Yu, Ziyi Shen, Chuanhui Gu, Yanchao Bai

**Affiliations:** 1 College of Environmental Science and Engineering, Yangzhou University, Yangzhou, 225127 People’s Republic of China; 2 Jiangsu Collaborative Innovation Center for Solid Organic Waste Resource Utilization, Nanjing, 210095 People’s Republic of China; 3 School of Environment, Beijing Normal University, Beijing, 100875 People’s Republic of China

**Keywords:** Biogeochemistry, Carbon cycle, Element cycles

## Abstract

The most important measures for salt-affected mudflat soil reclamation are to reduce salinity and to increase soil organic carbon (OC) content and thus soil fertility. Salinity reduction is often accomplished through costly freshwater irrigation by special engineering measures. Whether fertility enhancement only through one-off application of a great amount of OC can improve soil properties and promote plant growth in salt-affected mudflat soil remains unclear. Therefore, the objective of our indoor pot experiment was to study the effects of OC amendment at 0, 0.5%, 1.0%, 1.5%, and 2.5%, calculated from carbon content, by one-off application of sewage sludge on soil properties, rice yield, and root growth in salt-affected mudflat soil under waterlogged conditions. The results showed that the application of sewage sludge promoted soil fertility by reducing soil pH and increasing content of OC, nitrogen and phosphorus in salt-affected mudflat soil, while soil electric conductivity (EC) increased with increasing sewage sludge (SS) application rates under waterlogged conditions. In this study, the rice growth was not inhibited by the highest EC of 4.43 dS m^−1^ even at high doses of SS application. The SS application increased yield of rice, promoted root growth, enhanced root activity and root flux activity, and increased the soluble sugar and amino acid content in the bleeding sap of rice plants at the tillering, jointing, and maturity stages. In conclusion, fertility enhancement through organic carbon amendment can “offset” the adverse effects of increased salinity and promote plant growth in salt-affected mudflat soil under waterlogged conditions.

## Introduction

Arable land in China is scarce, and the per capita area of arable land is approximately 0.1 hm^2^ and less than 40% of the world average. With the increasing urban sprawl and economic development, the total area of arable land in China is decreasing and is gradually approaching the lower limit needed to ensure food security^1, 2^. Approximately 20,000 hm^2^ of mudflats form on the eastern coast of China every year^3^. After reclamation and reconstruction, these flats can be an important source of arable land. Over the past 50 years, China has added 1.1 to 1.2 million hectares of land through mudflat reclamation^4^. According to the current sedimentation rate of rivers entering the sea in China, another 1.0 to 1.5 million hectares of land is estimated to be reclaimed by 2050^5^. Coastal mudflat soil under new reclamation has poor soil structure, high salinity, low fertility, and poor microbial flora. Natural maturation and traditional improvement processes generally take 15 to 30 years to implement. The most important measures of salt-affected mudflat soil reclamation are to reduce salinity and to increase soil organic carbon content and thus soil fertility.

The process of salinity reduction can be accelerated by irrigation with freshwater and by concentrated rainfall. Planting rice to facilitate salt leaching is also an important measure for promoting rapid reductions in the salinity of mudflat soil. Rice is a wet-loving crop; thus, maintaining a flooded water layer in the field is imperative. The soluble salt in the soil layer is dissolved by water infiltration in the field and then carried by water and removed by drainage facilities, which leads to salinity reduction of the surface soil. This is an old but effective method for reducing the salinity of salt-affected soil^6^.

The key to rapidly improving soil fertility is increasing soil organic carbon (OC)^7, 8^. Sewage sludge is a byproduct of the process of domestic sewage treatment, which is considered both as “resources” and “pollutants”^9^. The organic matter content of sewage sludge can be as high as 40% to 50%. In addition, sewage sludge also contains a large amount of “resource”^10–12^, such as nitrogen and phosphorus, as well as “pollutants” such as heavy metals, refractory organics, persistent organics, and microplastics^13–16^. The treatment and disposal of sludge have become a subject of wide interest among researchers. With the gradual optimization of the urban sewage pipe network, the inorganic components of sewage sludge are expected to decline, and the organic components are expected to increase to even 60–70%^17^. These organic components can be used as a source of OC for the improvement of salt-affected soil on mudflats^8^.

The previous studies have confirmed that sewage sludge can be used as a source of OC to improve salt-affected soil in coastal mudflats, which can promote the growth and yield of plants, such as ryegrass and maize^18, 19^. However, whether fertility enhancement through one-off application of a great amount of OC can improve soil properties and promote plant growth in salt-affected mudflat soil under waterlogged condition remains unclear. We hypothesize that addition of sewage sludge will increase soil salinity due to the high salinity of sewage sludge, but increase in nutrients will offset the inhibition effect of increased salinity on rice growth. Here, we used OC from sewage sludge that adhered to agricultural standards (GB4284-2018) to study its effects on soil properties, rice yield, and root growth in salt-affected mudflat soil. Specifically, we used an indoor pot experiment under waterlogged conditions with varying amounts of sewage sludge (SS). Our findings clarify the mechanism by which organic carbon improves soil fertility on mudflats and provides a theoretical and practical basis by which mudflat soil can be rapidly improved.

## Materials and methods

### Experimental materials

The mudflat soil used was a typical salt-affected soil and sampled from the Fangling reclamation area (32°36′30″ N, 121°56′03″ E) in Rudong County, Jiangsu Province, China. The experimental rice cultivar was Huaidao 5 (*Oryza sativa* L.). The experimental pots were cylindrical plastic pots with a height of 40 cm and an inner diameter of 34 cm and sealed at bottom. The sewage sludge used for pot experiment was gathered from Chunguang Ecological Agriculture Development Co., Ltd. of Taizhou in Jiangsu province, and its quality complied with the China’s state standard for agricultural application of sewage sludge (GB/T 24,600–2009). The basic properties of mudflat soil and sewage sludge were shown in Table 1.

**Table 1:** Basic properties of the mudflat soil and sewage sludge used in this study.

Items	Mudflat soil	Sewage sludge
pH	8.74	6.04
Salinity (‰)	3.12	9.12
Organic carbon (g kg^-1^)	4.54	488.54
Total N (N g kg^-1^)	0.33	35.42
Total P (P g kg^-1^)	0.59	16.44
Alkaline N (N mg kg^-1^)	16.54	1689
Available P (P mg kg^-1^)	11.65	469

### Experimental design

The pot experiment was performed in the greenhouse of the College of Environmental Science and Engineering, Yangzhou University from May to October, 2018. The mudflat soils were air-dried, sieved through a 2-mm sieve, filled to 10 kg per pot, and soil bulk density in the pot was 1.3 g cm^−3^. The treatments of SS application were conducted by mixing the sewage sludge at the rates of 0, 160, 320, 480, and 800 g on a dried weight basis uniformly with 0–20 cm soil in the pots to reach 0, 0.5%, 1.0%, 1.5%, and 2.5% OC of soil weight in the 0–20 cm soil layer, respectively. Each treatment had nine pots for three replicates for three sampling times. All pots were completely randomly arranged in the experimental area of the greenhouse. The soils in each pot were submerged with 5 cm water layer above the soil surface. To offset evaporation, sporadic irrigation was applied to maintain the 5-cm water layer. A total of 0.75 g urea (N 46%), 0.40 g superphosphate (P_2_O_5_ 12%), and 1.33 g potassium chloride (K_2_O 60%) were used as basal fertilizer in each pot. On June 20, four rice seedlings of 3.5-leaf stage were selected and transplanted into each pot. The 0.375 g urea per each pot was applied as tiller fertilizer and ear fertilizer on July 4 and August 17, respectively. The root, aboveground parts, and bleeding sap of rice plants were collected at the tillering stage (July 20), heading stage (September 1), and maturity stage (October 20) from three pots at random of each treatment. The rice plants were harvested and sampled on October 30.

### Soil analysis

Soil samples for 0–20 cm depth were collected in triplicate from last three pots of each treatment on October 30. Soil OC was determined by the K_2_Cr_2_O_7_ external heating method. Soil electric conductivity (EC) and soil pH were determined by a 5:1 soil and water ratio of conductivity meter and pH meter, respectively. Soil total nitrogen (N) and total phosphorus (P) were measured using the semi-micro Kelvin method and the H_2_SO_4_-HClO_4_ digestion method, respectively. Alkaline N and available P were determined using the alkaline hydrolysis diffusion method and the NaHCO_3_ extraction-molybdenum antimony colorimetric method^20^.

### Plant analysis

Four rice plants were randomly selected to collect bleeding sap of rice plant, and the stem was cut off at a distance of 5 cm from the root. After 10 min, the tissue fluid at the broken stem was absorbed with filter paper. The broken stem was quickly wrapped with absorbent cotton so that the end face of the stem was in contact with it. The absorbent cotton was then wrapped in a plastic bag and collected 14 h later (18:00–8:00). The bleeding sap was measured using the weighing method, and the contents of soluble sugar and amino acids were determined using the anthrone and ninhydrin method^21^. After the bleeding sap was collected, the root of the rice plant was collected to measure root growth and root activity by the winrhizo2003b root analysis system and the TTC method^21^. The total root length, root surface area, average diameter, and total volume were calculated. The aboveground parts of rice plants were deactivated at 105 ℃ for 15 min, and oven-dried at 80 ℃ until constant weight was reached, and then weighed to obtain the biomass.

### Data analysis

A single-factor analysis of variance (ANOVA) with completely randomized design (CRD) was performed for the data obtained in the study and the least significant difference (LSD) method was used to detect significant difference between the treatments of sewage sludge application.

## Results and analysis

### Soil chemical properties of mudflats salt-affected soil

The SS application increased OC concentration of the mudflat soil, whereas pH decreased as compared with the unamended soil (Table 2). The concentration of total N and P, alkaline N, and available P in mudflat soils increased with increasing SS application rate. Compared to the control soil, there were increases in soil EC by 1.5%, 5.8%, 10.7% and 12.4% at 0.5%, 1.0%, 1.5% and 2.5% SS application rates, respectively.

**Table 2:** Chemical properties of salt-affected mudflat soil at different sewage sludge application rates.

Parameters	Sewage sludge application rates				
	0	0.5%	1.0%	1.5%	2.5%
pH (5:1)	8.43 ± 0.04a	8.31 ± 0.01b	8.20 ± 0.08b	8.08 ± 0.02c	8.05 ± 0.04c
EC (dS m^-1^)	3.94 ± 0.11b	4.00 ± 0.11b	4.17 ± 0.04ab	4.36 ± 0.08a	4.43 ± 0.13a
Organic carbon (g kg^-1^)	4.53 ± 0.20e	5.23 ± 0.13d	6.73 ± 0.32c	8.81 ± 0.33b	12.82 ± 0.30a
Total N (N g kg^-1^)	0.44 ± 0.01e	0.63 ± 0.02d	0.82 ± 0.09c	1.03 ± 0.00b	1.53 ± 0.08a
Total P (P g kg^-1^)	0.43 ± 0.05e	0.63 ± 0.02d	0.75 ± 0.04c	0.92 ± 0.01b	1.05 ± 0.05a
Alkaline N (N mg kg^-1^)	45.47 ± 0.91d	49.75 ± 0.96d	72.05 ± 1.27c	95.89 ± 7.30b	123.48 ± 5.91a
Available P (P mg kg^-1^)	25.40 ± 2.37d	32.73 ± 1.46d	64.33 ± 1.64c	77.00 ± 3.24b	133.67 ± 7.88a

Values are mean ± SD of three replicates. Different letters in each row indicate significant difference at *p* < 0.05 by LSD’s multiple range test.

### Biomass and yield of rice plants

**Figure 1:**
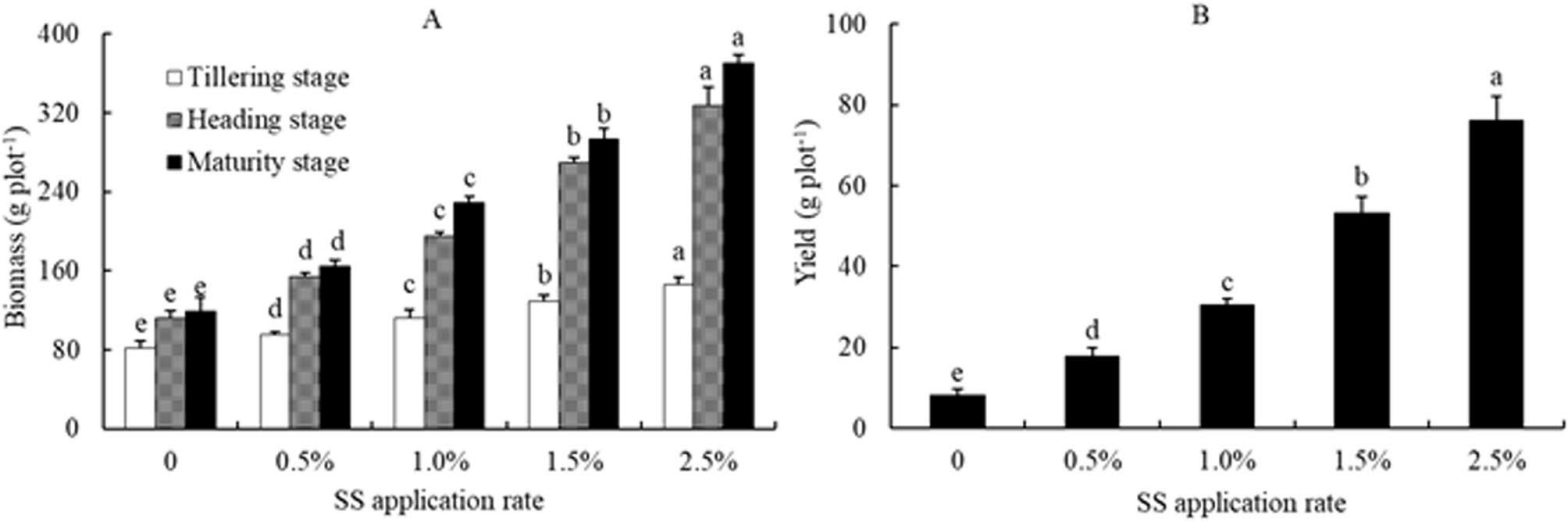
Effect of sewage sludge (SS) application on rice aboveground biomass (**A**) and yield (**B**) in salt-affected mudflat soil. Columns with different letters show significant difference between SS application rates at *p* < 0.05 by LSD’s multiple range test.

The effects of sewage sludge application on biomass and yield of rice plants are shown in Fig. 1. The aboveground biomass of rice plants was affected by the application of sewage sludge, the highest value occurring for the 2.5% SS application rate, and the lowest for the unamended soil. The grain yield of rice increased by 114.4%, 267.3%, 537.1%, and 811.7% at 0.5%, 1.0%, 1.5%, and 2.5% SS application rates, respectively, compared to the control.

### Root growth and activity of rice plants

**Figure 2:**
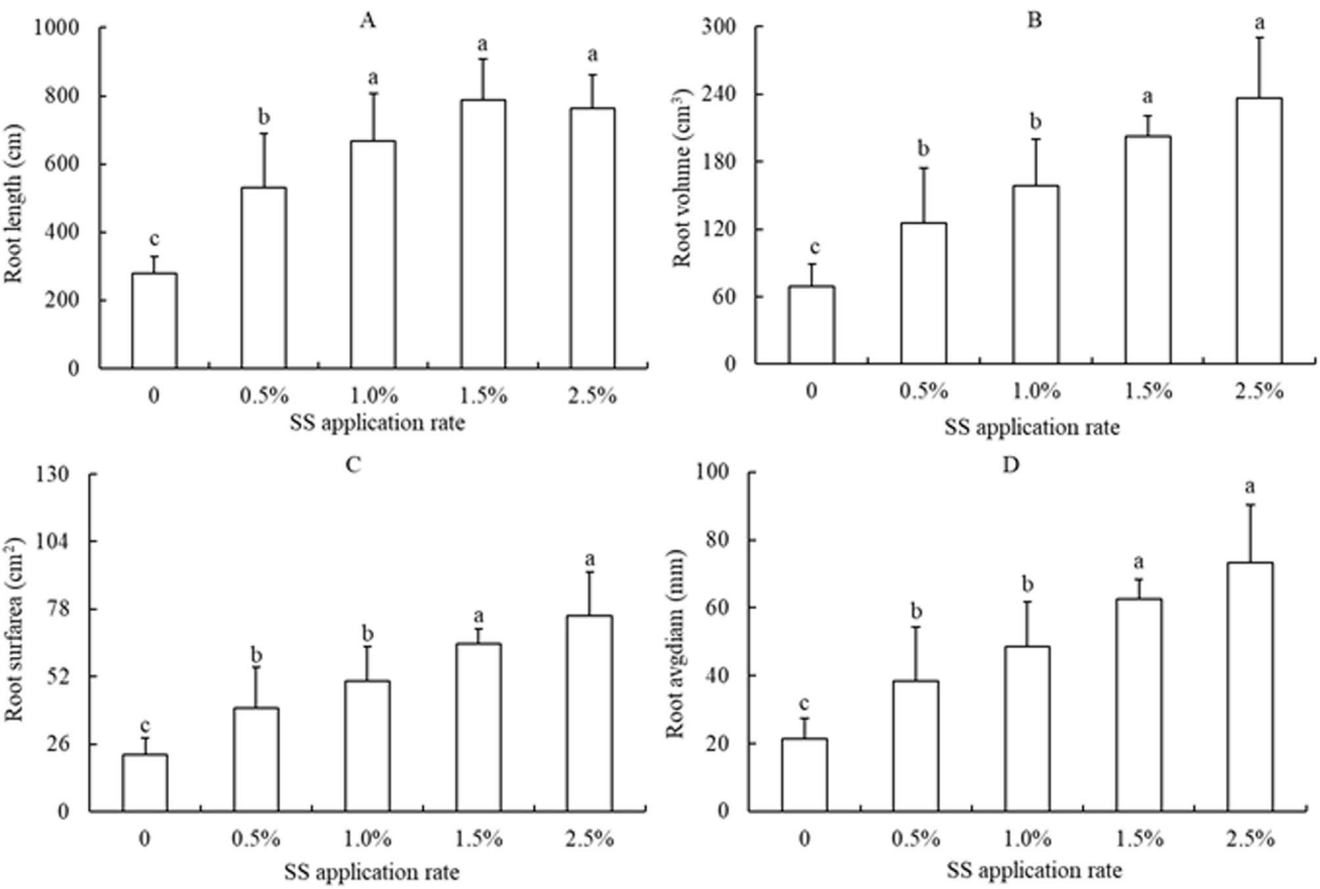
Effects of sewage sludge (SS) application on root length (**A**), root volume (**B**), root surface area (**C**) and root diameter (**D**) of rice plants at tillering stage in salt-affected mudflat soil. Columns with different letters show significant difference between SS application rates at *p* < 0.05 by LSD’s multiple range test.

The total root length, total volume, root surface area, and average diameter of rice roots increased with increasing SS application rate (Fig. 2). The total root length, total volume, root surface area, and root diameter of the rice plants at 2.5% SS increased by 467.0%, 393.9%, 588.5%, and 25.2% respectively, compared to the control.

**Figure 3:**
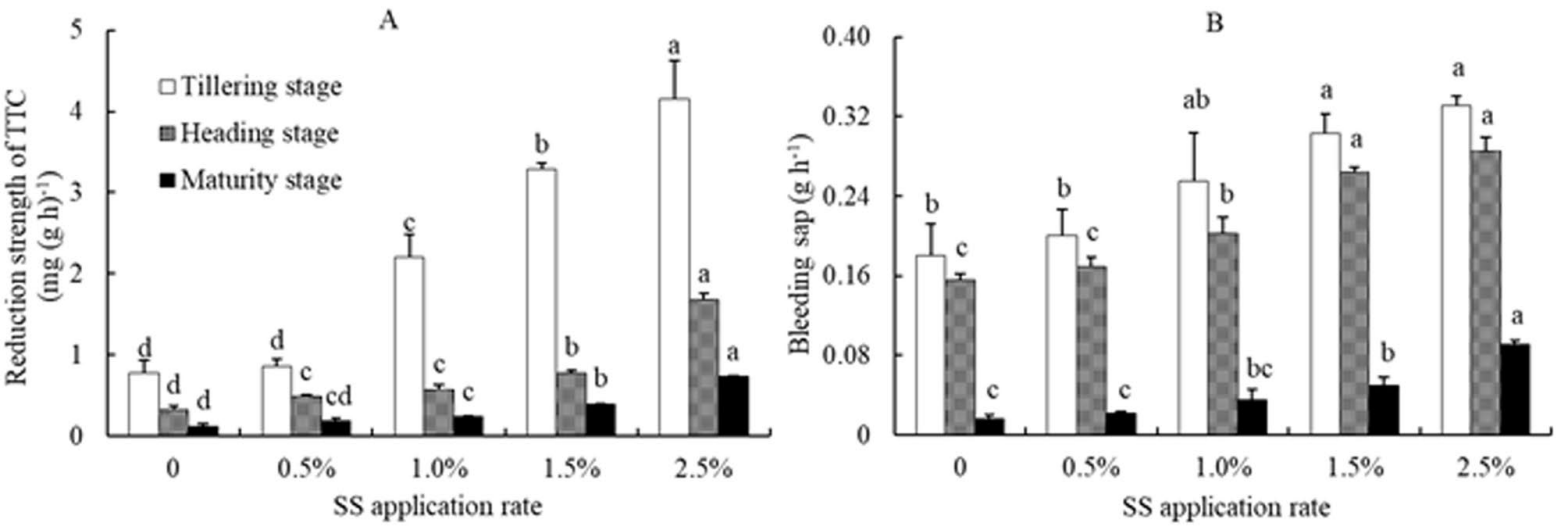
Effects of sewage sludge (SS) application on root activity (**A**) and bleeding sap (**B**) of rice plant in salt-affected mudflat soil. Columns with different letters show significant difference between SS application rates at *p* < 0.05 by LSD’s multiple range test.

The effects of SS application on root activity and flux activity of rice plants were shown in Fig. 3A,B. The root activity and flux activity of rice plants increased with increasing SS application rates during each growth stage. At the tillering stage, the root activity and the flux activity of rice plants increased by 12.2%, 185.8%, 325.3% and 438.0%, and by 11.2%, 41.4%, 67.6% and 83.1%, at SS application rates of 0.5%, 1.0%, 1.5%, 2.5% , respectively. At the jointing and maturity stages, the root activity and flux activity of each treatment were the same as those of the tillering stage.

### Soluble sugar and amino acid in bleeding sap of rice plants

**Figure 4:**
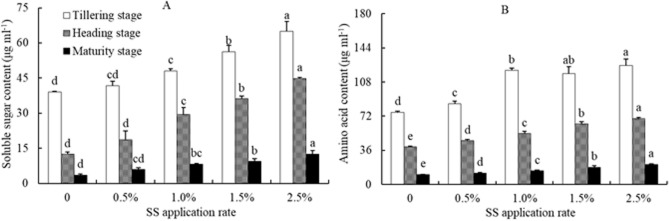
Effects of sewage sludge (SS) application on the concentration of soluble sugar (**A**) and amino acid (**B**) in the bleeding sap of rice plant in salt-affected mudflat soil. Columns with different letters show significant difference between SS application rates at *p* < 0.05 by LSD’s multiple range test.

The concentration of soluble sugar and amino acid in the bleeding sap of rice plants at different growth stages increased as the amount of SS applied increased (Fig. 4-A and B). At the tillering stage, the contents of soluble sugar and amino acid at 0.5%, 1.0%, 1.5%, and 2.5%SS application rates were 6.9%, 22.9%, 43.9% and 65.9%, and 12.0%, 58.0%, 53.6% and 64.6% higher than those in the control. At the jointing stage and maturity stage, the contents of soluble sugar and amino acid in rice plants bleeding sap of each treatment were similar to those at the tillering stage.

## Discussion

The application of SS increased the OC content and reduced the pH in mudflat salt-affected soil under waterlogged condition. The low pH of sewage sludge and the intermediate products, such as organic acid, produced by incomplete decomposition after entering the mudflat soil may explain the decrease in pH of the mudflat soil^22^. The OC in the sludge entering the mudflat soil was not mineralized easily under waterlogged conditions. Thus, the OC in sludge is more susceptible to humification and conversion to mudflat OC. Our previous studies have shown that the application of sewage sludge significantly increased soil OC in salt-affected drylands in mudflats^8, 23^. Previous studies have found that the application of SS significantly increased the OC content of calcareous alluvial soil^24^, acid mine soil^25^, and vineyard sandy soil^26^.

The concentration of nitrogen and phosphorus nutrients in mudflats salt-affected soil showed an increasing trend with increasing SS application rate. The application of sewage sludge rich in N and P nutrients significantly increased the soil fertility of salt-affected drylands on mudflats^23^. The previous studies also show that the application of SS can improve the nutritional status of farmland soils^27–29^. However, the rates of increase varied among these elements. Total and alkaline N in the soils treated with SS increased by 41.2–244.3% and 9.4–171.6%, respectively. Total and available P increased by 47.4–147.0% and 28.8–426.2%, respectively. The N and P nutrients in the sludge primarily exist in organic forms, which cannot be transformed into available nutrients until they enter the soil and become gradually mineralized^27^. Different types of soil and different soil environmental conditions affect the mineralization rate of organic matter^28^. Under water-logging condition, the application of SS was inclined to accumulation of total N and available P in the salt-affected mudflat soil. Low organic matter mineralization rate under water-logging condition and large plant uptake of soil available N may explain why the total N accumulation ratio was greater than the available N ratio in the salt-affected mudflat soils. Given the corresponding N and P contents of mudflat soil and sewage sludge, the N and P absorbed by rice plants, and the input of N and P from fertilization, large amounts of N may be released into the atmosphere through denitrification. Flooding conditions are conducive to improving the availability of soil P, mainly due to elevated solubility and reduced fixation of soil P induced by the changes of pH, redox and intermediate organic matters during soil flooding^30^. These are important reasons why the accumulation ratio of available P was greater than the total P ratio in the salt-affected paddy mudflat soil.

The application of SS increased the soil EC of salt-affected mudflat soil under waterlogged conditions. This increase raised from the higher salinity in sewage sludge than that in the mudflat soil. However, studies on salt-affected dryland improvement under the natural conditions have shown that the application of SS reduced soil salinity in contrast to the findings of this study^7^. This discrepancy can be explained by the fact that the SS entering the mudflat soil can improve the agglomeration of soil particles, promoted the formation of > 0.25 mm soil aggregations^31^, thereby reduced soil bulk density, increased non-capillary porosity, increased downward channel of water and salt, reduced capillary rise of water and salt, and therefore inhibited return of salt^32^. However, in this study, the salt entering the soil did not have a downward leaching channel and can only be stored in the test pot, thereby increased the soil salinity (as measured by EC).

The growth of rice plants was not inhibited by the increase in soil salinity, and rice biomass and grain yield increased as more sludge was applied. Rice plants can tolerate soil salinity contents up to 4–6 dS m^−1^^33, 34^. Nevertheless, the soil salinity of the treatments was 4.00–4.43 dS m^−1^ in this study, which is close to the upper limit of the salt tolerance of rice plants. However, the growth of rice plants was not affected by the increase in salinity. The application of SS might have indirectly increased the salt tolerance of rice plants through promoting the growth of rice roots. Root growth status and the regulation of root osmotic potential are critical factors for determining salt tolerance^35^. The application of SS promoted the growth of rice roots and increased the total root length, root surface area, root diameter, and root volume of rice roots. In addition, root activity also increased as the amount of sludge applied increased. Previous studies have also confirmed that the application of SS can promote the root growth of sunflower^36^ and ryegrass^37^. Demir et al. studied maize plants and showed that the application of sludge can promote maize root growth and increase root biomass^38^. Other studies have found that the application of sewage sludge can increase the root vitality of rice^39^, and ryegrass^37^. The soluble sugar and amino acid contents in the bleeding sap of the plant roots are important indicators of the root penetration potential of the plant^40, 41^. This study confirmed that the application of SS increased the soluble sugar and amino acid content in the bleeding sap of rice roots. This finding may also explain why rice growth and grain yield are not affected by increasing soil salinity content.

## Conclusions

The application of SS promoted soil fertility by elevating nutrient content and improving chemical properties of mudflat soil under waterlogged condition. SS application promoted the root growth of rice plants thus enhanced the tolerance of the rice to salt stress. Our results suggest that fertility enhancement only through one-off application of a great amount of sewage sludge might improve soil properties and promote plant growth in salt-affected mudflat soil.
